# Cortisol and Secretory Immunoglobulin A Response to Stress in German Shepherd Dogs

**DOI:** 10.1371/journal.pone.0090820

**Published:** 2014-03-17

**Authors:** Ivona Svobodová, Helena Chaloupková, Roman Končel, Luděk Bartoš, Lenka Hradecká, Lukáš Jebavý

**Affiliations:** 1 Czech University of Life Sciences Prague, Department of Animal Science and Ethology, Praha 6 – Suchdol, Czech Republic; 2 Institute of Animal Science, Department of Ethology, Praha Uhříněves, Czech Republic; GI Lab, United States of America

## Abstract

The aim of the study was to determine whether cortisol and secretory Immunoglobulin A (sIgA) could be used as an indicator of acute stress in both young and adult dogs. Seventeen German shepherd puppies were exposed to the Puppy test (challenge test) at the age of seven weeks. This test has been routinely used to assess the future working ability of potential police dogs. In addition, ten adult females were subjected to 4 minutes of defense training under stressful conditions. Saliva was collected from the puppies and adult females before testing and 20 minutes after the start of testing, using a cotton swab held for 1–2 minutes in each dog's mouth. Cortisol concentrations increased after the test compared to the control sample both in puppies and the adult females. However adult females showed a significant decrease in sIgA after defense training while puppies showed a tendency of increase in sIgA. We propose that salivary cortisol could be used as an indicator of stress in puppies during early ontogeny. It is not yet clear whether sIgA could be used as a useful indicator of short-term stress in dogs.

## Introduction

There is an increasing need for specialized working dogs in human society (e.g. personal detection, detection of dangerous substances, guide dogs, etc). Dogs are exposed to many challenging situations such as traffic, unusual distractions including noise, contacts with unfamiliar persons and other animals including dogs. It is necessary to select and train dogs with appropriate personal characteristics - most importantly the ability to cope with stressful situations, which is crucial for effective training as well as their welfare [1], [2].

Behavioral assessment of the working potential of dogs is a frequently used tool in the selection of suitable dogs for guide, police, and military work [2], [3], [4]. These assessments are based on measuring behavioral reactions towards various startling stimuli, novel situations, tactile/audio stimuli and willingness to fetch. Selection of the puppies for police work was done in accordance with the results of the tests, thereby helping to improve overall training results and identify methods for early detection of dogs with exceptional working potential [5], [6], [7]; factors which can help to reduce the cost of training. However, the predictive value of puppy tests has remained controversial and the results are not clear.

Behavioral characteristics are linked to different physiological and neuroendocrine responses to threatening situations [8], [9] and are consistent over time. Some studies provided in non-canine species found that after stress tests there is a change in both behavior and physiology [10], while other studies found changes only in physiology or only in behavior [11], [12]. This suggests that behaviour and stress physiology in some cases may operate independently of each other. Therefore, the need for research focus on physiological stress responses is important, mainly during early ontogeny. Cortisol is probably the most frequently measured indicator of stress in various domestic animals such as cows and pigs [13], [14], horses [15], sheep [16], etc. In adult dogs, cortisol was measured in plasma [17], urine [18], saliva [19], feces [20] and hair [21]. Salivary cortisol has been shown to be a useful non-invasive measure of acute and chronic stress in dogs [22] and has a direct correlation with plasma concentration. In adult dog saliva, the peak cortisol level was measured 20 minutes after the start of the acute stress test [23], but salivary cortisol response to acute stress during early ontogeny of dogs has not yet been fully tested and there is a question, whether cortisol measurement could be useful marker for puppy assessment of stress.

Recently, other possible non-invasive methods for measuring stress is salivary Immunoglobulin A (sIgA). IgA is the most abundant class of antibody in mucous membranes, where it is an essential factor in protecting against infectious agents, allergens and foreign proteins [24] and has a concentration which could be affected by stress. It has been found that levels of sIgA in adult dogs decreased following long-term stress [25] and an acute stress [26]. Several studies have provided analyses of sIgA in humans. They suggest that sIgA increases after short-term stressors [27], [28], however one study showed a decrease [29]. Nevertheless, some previous studies detected the relationship between cortisol and sIgA levels after stressful situation in adult dogs: [1], [6], [22], [25], [30], [31] and also in children [32]. In children higher concentration of cortisol related with lower concentration of sIgA and together these associations had relationship with higher incidence of illness [32]. Therefore, it is not clear whether also sIgA could be used as an indicator of acute stress and is related with cortisol concentration in puppies. Measurement of the physiological responses to acute stress in puppies could help to assess suitable dogs for future use and could also be useful in the assessment of the welfare of working dogs. The aim of this study was to determine (i) whether salivary cortisol is a suitable indicator of acute stress (ii) whether salivary sIgA concentrations change before and after a stress situation, and (iii) whether sIgA shows any relationship with cortisol and could thus be used as an alternative indicator of acute stress.

## Materials and Methods

### Ethics Statement

This study was carried out in strict accordance with the recommendations in the Guide for the Care and Use of Animals of the Czech University of Life Sciences Prague. The protocol was approved by the Czech Central Committee for Protection of Animals (Permit number: MŠMT 26663/2010-30, 7/2010). All manipulations were made to minimize suffering. The Czech Republic Police Breeding Facility as owner of the German shepherd dogs gave permission for their animals to be used in this study.

### Animals and housing

All procedures involving animals were approved by the Animal Care and Use Committee of the Czech University of Life Sciences and Ministry of Agriculture (Prague, Czech Republic).

The German shepherd puppies to be tested were kept at the Czech Republic Police Breeding Facility (CRPBF), Domažlice, Czech Republic, in 2010 and 2011. In total, 17 puppies (9 males and 8 females), aged 7 weeks from seven litters (mean 6 puppies per litter) from six mothers. Then ten adult females, unfamiliar with puppies, at the age of 3.33±1.77 years (mean±S.E.) were included in the study and were tested during anestrus or metestrus.

All dogs were kenneled in large fence enclosures with doghouses. Puppies were weighed once a week until the age of six months. They were dewormed at 8–9 days, and again at 3, 5, and 7 weeks, when they were weaned. Individual puppies were identified by unique fur cut and at 7 weeks were tattooed and micro chipped. Doghouses and kennels were regularly washed with hot steam and disinfected with SAVO, Kresolan or Desam OX. Kennels were cleaned at least twice a day and pens once a week.

During the first three weeks post-partum, human contact with the puppies was limited to treatment, weighing, feeding of mothers and cleaning of the delivery area of the kennels. From age 3 to 7 weeks the puppies were walked outside the kennels without their mothers.

### Experiment design

#### Puppy test

This specific puppy test has been routinely used for more than 20 years to assess the future working ability of police dogs in the Czech Republic. The puppies are exposed to ten short stressful tasks which include: exposure to a new environment, a new person, a new object, and noises. For the description of the complete Puppy test see Svobodová et al. [4].

All pups were tested separately from other conspecific before morning feeding at the age of 7 weeks ± 1 day. After completion of the ten minute test, puppies were returned to their home environment. The results from the puppy test were not finally included to the study because 8 from 10 tasks showed no variability among these puppies and therefore it makes no sense to analyze it statistically.

#### Defense training of adult dogs

Ten adult female dogs were tested during a one day period in the training site familiar to them. Each female was individually trained with one handler and had no visual contact with other tested females. A figure rushed out from behind the screen from a distance of approximately 5 m. After a short provocation, done by cracking the whip and vocalizing loudly, he started moving towards the dog, waving the arm protection sleeve from a distance of 3 m, and gave the sleeve to the female when she got hold of it. The figure then walked one or two paces, dragging the dog, and continued to threaten it by striking the whip-handle on the leash held by the handler. The figure dropped the sleeve to the dog after one minute and then several seconds later the handler recovered the sleeve and returned it to the figure. The defense training was repeated once again in the same manner.

### Saliva sampling and analysis

The puppies were sampled before and after the behavioral puppy test, while the adult dogs were sampled before and after the defense training. Control saliva samples were taken 15–30 min before testing in the home environment. Testing and the consequent sampling were consistently done in the morning between 7–11am and the exact time the sample was taken was recorded.

The saliva for analysis was collected on a cotton swab 20 min after the start of the stress situation when salivary concentration was at its peak [23]. A clean swab was inserted and held in the mouth of each dog for 1–2 minutes, placed in a plastic tube and then frozen. After thawing the samples in the laboratory, they were centrifuged and analyzed.

The concentration of dog salivary cortisol was determined using the Enzyme Immunoassay KIT (# 1-3002, Salimetrics Europe, Ltd., UK). This competitive assay is based on a monoclonal antibody coated in microtitre plate and cortisol linked to horseradish peroxidase. Specification of cross-reactivities of the cortisol antibody with several endogenous and pharmaceutically employed steroids were provided by the manufacturer (Salimetrics Europe, Ltd., UK). The intraassay and interassay CV were 3.55 and 6.41%, respectively. Recovery of cortisol added to saliva was between 96.9 and 106.1% for doses of 0.1, 3, 20, and 25 ng of cortisol/mL. The minimum detectable concentration of the assay was 0.03 ng/ml. The concentration of dog IgA in saliva samples was determined using the Dog IgA ELISA Quantitation KIT (# E40-104, Bethyl Laboratories, INC., USA). The standard and sample were incubated in wells of microtitre plate coated with goat anti dog IgA antibody in the first step. After washing off non-binding protein, the conjugate of goat anti dog IgA antibody with horse radish peroxidase was incubated. Finally, after washing off non-binding conjugate, TMB colorimetric substrate was added and colorimetric reaction was stopped by using 0.2 M H_2_SO_4_. Due to the high content of dog IgA in saliva, each sample was diluted before analysis by sample diluent, using a dilution factor of 1000 and 5000. Read off values were multiplied by the dilution factor; the mean of both values is presented in the recorded stated results.

### Statistical analyses

All data were analyzed using SAS (version 9.2). Results were considered statistically significant when P ≤ 0.05. The differences in cortisol and sIgA concentrations before and after the challenges (Puppy test and defense training) were tested using the Generalized Linear Mixed Models (PROC MIXED, further GLMM) with repeated measures design (subject  =  challenges before and after). To account for the repeated measures on the litter, the analysis was performed identity of the litter as a random effect. The significance of each fixed effect in the GLMM model was assessed by the F-test. All analyses were performed with the individual dog as a statistical unit. Cortisol and sIgA concentrations were log-transformed to improve normal distribution (normality tested by Kolmogorov-Smirnov test).

#### The effect of stress on the cortisol and sIgA concentrations

Cortisol and sIgA concentrations were used as the dependent variables; fixed effects were class “sampling order” (before and after tests), class “sex” (male or female), class “age category” (puppy or adult), interaction “sampling order” * “age category” and the continuous variables: “body weight”, “time of sampling” and “age” were the fixed factors. Non-significant effects were dropped from the model and will not be mentioned in further text.

#### The relationship between differences in the cortisol and sIgA after stress

Firstly the new variables were counted: as “cortisol difference” and “sIgA difference” (we calculated “before test/defense” minus “after test/defense”). Secondly, PROC MIXED analyzed the relationship, where “sIgA difference” was the dependent variable and ”cortisol difference” was the continuous fixed effect with “age category” (puppy vs. adult) as the categorical fixed effect.

Least square means were calculated by computing the mean of each treatment (class) and averaging the treatment (CLASS) means. These means of means are then used to compare the factors. In this way, the means are adjusted for the number of observations in each treatment. This estimate is unbiased because of the unequal number of observations taken into account. Least-squares means (also referred to as “adjusted means”) were computed for each class and differences between classes were tested using the t-test. The Tukey-Kramer adjustment was used for multiple comparisons.

For all models we have assessed the normality of residual effects. Because the p-value in all cases was greater than 0.15, the hypotheses of normality was not rejected.

## Results

### Cortisol and sIgA after acute stress

**Figure 1:**
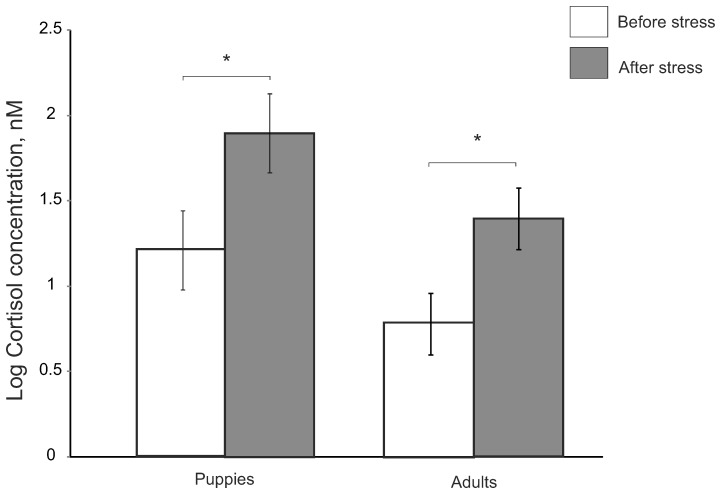
Salivary cortisol concentration of puppies and adults taken before acute stress and 20 min after the start of acute stress after logarithmic transformation (adjusted means ± S.E.). ^*^P<0.05.

Cortisol concentration after the acute stress increased compared to the control sample in both, puppies and adults (F_1,22_ = 20.91, p<0.001, Fig. 1). Adult females tended to have lower concentration of cortisol than puppies (F_1,22_ = 3.05, p<0.10).

**Figure 2:**
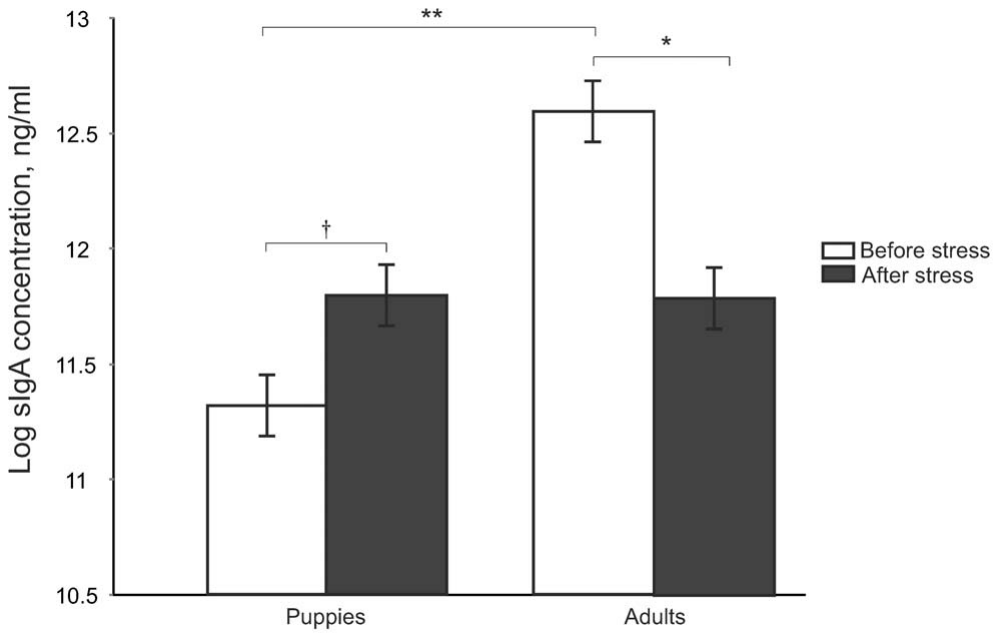
sIgA concentration of puppies and adults taken before acute stress and 20 min after the start (adjusted means ± S.E.). ^†^P<0.1, ^*^P<0.05, ^**^P<0.01.

Salivary IgA was significantly affected by the acute stress in an interaction with the age category (F_1,34_ = 12.95, p<0.001). While puppies tended to have higher sIgA after the stress, it was just opposite in adults whose sIgA decreased after the stress (Fig. 2). No other fixed effect affected the cortisol or sIgA concentrations after the acute stress.

### The relationship between differences in the cortisol and sIgA after stress

No significant relationship between cortisol and sIgA differences was found (F_1, 3_ = 0.15; P = 0.72).

## Discussion

In accordance with reports on adult dogs and our results on adult females, the current pilot study has supported the suggestion that salivary cortisol concentration can also be used as one indicator of acute stress in puppies at seven weeks post-partum. This is in contrast to the results of Palazzolo and Quadri [33], who did not find any cortisol responses to stress from low ambient temperature (−5°C) in puppies at the age of eight weeks compared to adult dogs. However, no clear result in the measurement of the sIgA was found. In puppies, a tendency to increase sIgA was detected while in adult females, in response to an acute stress, there was a decrease in sIgA concentration as suggested by Kikkawa et al. [26].

In previous studies other authors reported that concentration of the fecal IgA was lower in 5-month-old puppies compared to 4-year-old adults [24], which was also found in the present study. However, in the present study the puppies were aged 7 weeks only. In young puppies, IgA has been detected in nasal secretions and serum in neonatal puppies only [34]. Also, in humans, salivary IgA levels increased with age of children. Salivary IgA were not measurable during the gestation period and were detected till 4–6 weeks after birth and continued to increase up to the age of 18 months [35]. Although the current results might be promising, still for the time being sIgA cannot be recommended as an indicator of acute stress for young puppies. SIgA concentrations increased after the stress, while in adults decreased. It is suspicious that the sIgA concentrations revealed no relationship with the cortisol concentrations, which tended to increase in both, puppies and adults. Yet, the fact that cortisol concentrations tended to be higher in the puppies than in adults is in agreement with other study [36].

Although our results with adult females are in agreement with the study of 12-month old beagles, in which noise was used as a stress [26], we are still not convinced that this is a sufficient argument for accepting sIgA as an alternative indicator of stress in dogs because there is little general information about sIgA in dogs [26]. From other species, studies focused mainly on humans. SIgA increased after stress rather than decreased (e.g., [37], [38]). Similarly, increased sIgA was found in 6–8 weeks old piglets after short restraint [39]. It has been reported that the immune system distinguishes between natural and specific immunity, where Secretory IgA comes under the specific immune system [40]. A meta-analysis of human studies [40] showed that acute stressors (for duration of several minutes) were associated with potentially adaptive increased regulation of some parameters of natural immunity and decreased regulation of some functions of specific immunity. However, one exception was a significant increase of sIgA which was probably due to release of an already-synthesized antibody from plasma cells and increased translocation of an antibody across the epithelium and into saliva [41]. This effect therefore represents relocation, albeit of an immune protein rather than an immune cell.

Chronic stress may elicit prolonged secretion of cortisol, to which white blood cells mount a counter regulatory response by down regulating their cortisol receptors. This down regulation, in turn, reduces the cells' capacity to respond to anti-inflammatory signals and allows cytokine-mediated inflammatory processes to flourish [42]. However, the relationship between cortisol and sIgA after short-term stress is not clear. The present study did not show any significant relationship between changes in the cortisol and sIgA in dogs. Positive correlation in humans was reported after a short term arithmetic task [37] and during intensive training periods in male weightlifters [43] and negative correlation in adult human after awakening [44], in adult students after repetitive examinations [45], [46] and also in pre-school children during child care days [32]. Thus it is not yet clear, whether sIgA could be used as a useful indicator of short-term stress in dogs.

In conclusion, our recent results suggest that unlike the sIgA concentration, measurement of the salivary cortisol concentration can be useful for indicating short-term stress in young puppies. Although adult females showed increased cortisol and decreased sIgA, no significant relationship between these two compounds was found. Further research in dogs is needed to clarify function of sIgA after short-term stress.
